# PREDICTORS OF INDIVIDUAL TRANSPORTATION ALTERNATIVES IN THE TRANSITION TO NON-DRIVING

**DOI:** 10.1093/geroni/igac059.3005

**Published:** 2022-12-20

**Authors:** Kellia Hansmann, Carolyn McAndrews, Stephanie Robert

**Affiliations:** William S Middleton Memorial Veterans Hospital, Madison, Wisconsin, United States; University of Wisconsin Madison, Madison, Wisconsin, United States; UW-Madison, Madison, Wisconsin, United States

## Abstract

For older adults who transition to non-driving, successful coping includes maintaining mobility. Individual transportation alternatives, e.g. rides from friends and family, are important coping factors in the transition to non-driving. However, it is unclear to what extent social network composition predicts the accessibility of these individual transportation alternatives. We examined whether various aspects of social networks (including living with a spouse/partner; having a child; living with a child; having other household members; having anyone to talk to about important things) were associated with the actual receipt of rides from family and friends. Using the National Health and Aging Trends Study (NHATS), a nationally representative cohort of Medicare beneficiaries ages 65 and older, we conducted a cross-sectional analysis using a subsample of community-dwelling older adults whose driving status was known at the baseline interview in 2015 (n = 5,889). We used logistic regression to estimate the association between social network indicators with the odds of receiving a ride from a family member or friend. When adjusting for biopsychosocial characteristics and current driving status, we found that having a spouse or partner was associated with a significant odds of having received a ride (adjusted OR, 95% CI = 1.41, 1.05–1.90) as was being able to name at least five people the participant could talk to about important things (adjusted OR, 95% CI = 1.65, 1.14–2.38). Our findings support previous evidence that marital status and support from social network members outside one’s household may be particularly important for accessible individual transportation alternatives.

